# Knowledge, attitudes, practices and perceived barriers towards research in undergraduate medical students of six Arab countries

**DOI:** 10.1186/s12909-022-03121-3

**Published:** 2022-01-18

**Authors:** Ahmed Assar, Sajeda Ghassan Matar, Elfatih A. Hasabo, Sarah Makram Elsayed, Mohamed Sayed Zaazouee, Aboalmagd Hamdallah, Alaa Ahmed Elshanbary, Asmaa Khaled, Helmy Badr, Hanan Abukmail, Khaled Mohamed Ragab, Shaimaa Sherif Soliman, IMedRA team of collaborators, Hamel Asma, Wiame Benhabiles, Imane Sahraoui, Boutheyna Drid, Imane Bakhtaoui, Nadia Hamidi, Mississilia Boulemssamer, Nour Salem, Yazan Omar Alawneh, Sief-Addeen Ziad Al-tahayneh, Malak Eyad Abu Qaddoura, Hala Aladwan, Obada Ahmad Obada Ahmad, Khulood Nasr, Mahmoud Aref Aref Aldrini, Nataly Mazen Mazen Salhab, Omar A. A. Safarini, Sami Dia, Sadi yehiankhala, Yousef Maher Maher Abuiriban, Nataly Mazen Mazen Salhab, Mohammed Al-kfarna, Rasha Mansour, Maria Nabil Nabil Alfathi, Rania Moh Hafez Mahfoud, Sami Jomaa, Mais Amin Amin Ibrahim, Abd Shbani, Rand Safwan Safwan Younes, Abeer Hassan Hassan Alkodsi, Mohammad-Nasan Abdul-Baki, Alma Douedari, Mai Deyaeldin Mohamed Mahmoud, Mona muhe eldeen eshag AbdAlrhman, Nosuiba Hamad Jumaa Mohamed, Delas Hussain, Mohamed Marey yahya Hassan, Noha Ahmed Ahmed Ammar, Marwa Abdelazim Abdelazim Rizk, Hossam Aldein Samir AbdElazeem, Ahmed Essam Helmy Mohammed, Shaimaa Abdelbadea, Hussien Saad Saad el-Ansarey, Mariam Ahmed Ahmed Maray, Ahmed Sultan, Ahmed Farag, Manar Hamdy Mohammed, Maryam abd elmalak shafik, Mohamed Essam, Asia Hamdy, Karim Usama, Yara Sakr

**Affiliations:** 1 International Medical Research Association (IMedRA), Al-Azhar University, Cairo, Egypt; 2 Faculty of Medicine, Menoufia University, Shebin El-kom, Egypt; 3 Faculty of Pharmacy, University of Jordan, Amman, Jordan; 4 Faculty of Medicine, University of Khartoum, Khartoum, Sudan; 5 Faculty of Medicine, October 6 University, Giza, Egypt; 6 Faculty of Medicine, Al-Azhar University, Assuit, Egypt; 7 Faculty of Medicine, Al-Azhar University, Damietta, Egypt; 8 Faculty of Medicine, Alexandria University, Alexandria, Egypt; 9 Faculty of Medicine, Tanta University, Tanta, Egypt; 10 Faculty of Medicine, Islamic University of Gaza, Gaza, Palestine; 11 Faculty of Medicine, Minia University, Minia, Egypt; 12 Faculty of Medicine, Menoufia University, Shebin El-kom, Egypt

**Keywords:** Knowledge, Attitudes, Practices, Undergraduate research, Medical research

## Abstract

**Background:**

The involvement of the undergraduates in the research field requires a better view of their potential and the anticipated barriers facing them. This study aims to assess the undergraduates' knowledge, attitudes, practices and perceived barriers towards research in six Arab countries.

**Methods:**

A cross sectional study included medical students from six Arab countries, where a self-administered five-section questionnaire was used to assess the students' demographics, knowledge, attitudes, practices and perceived barriers. The questionnaire was distributed in the online educational platforms of the participating medical schools in the six included countries.

**Results:**

The total sample of recruited students was 2989, the majority of students (91.6%) showed poor level of knowledge regarding research. Generally high levels of positive attitudes towards research, research relevance and usefulness were found, with moderate levels of perception of research anxiety and difficulty. 33.7% (*n* = 1006) participated in an actual research project before with a mean of .5 publications per student. Cross-sectional studies were the most common type of studies conducted by students (38.6%), followed by case reports (23.9%). Lack of access to lab equipment for lab research (68.1%), the priority of education over research (66.8%), and lack of time because of educational tasks (66.1%) were generally the top perceived barriers towards research practice.

**Conclusion:**

In the current study, the participants showed a poor knowledge level with associated positive attitudes towards research. One third of the students participated in research projects that mostly were cross-sectional studies and case reports. Educational tasks and lack of support were the most prevalent barriers. The students' positive attitudes towards research need to be translated into better knowledge and appropriate practice, which can be done by development of better training systems and more structured mentoring.

**Supplementary Information:**

The online version contains supplementary material available at 10.1186/s12909-022-03121-3.

## Background

Research is the most reliable way to expand scientific knowledge and improve the health care delivery. It is one of the best indicators to scientific advancement of a country [1]. Medical research is also essential for critical thinking and reasoning skills of a health professional [2]. Arab countries carry out little organized research compared to western countries. A clear lag in the medical research contribution can be noted in the area [3]. A study reported that the institutions in the Arab world published few researches and the majority of the published research papers were of poor quality [4].

The participation of medical students in research projects have been shown to influence the rate of publication in medical faculties [5], the thing which proposes undergraduates participation as a potential solution in improving the research situation in Arab countries. The potential of medical students lies in their ability to work as research assistants helping in research conduction, and on the long term, working as physician-scientists fulfilling the growing need for physician-scientists [6–8]. Also, Students' involvement in research during their study years helps in increasing their experience, and habits of scientific and critical thinking, which in turn, increases their research productivity later during their medical career [9–11].

An initial step in enhancing the undergraduates' role in the medical research field is exploring their underlying potential (knowledge and attitudes) and the anticipated barriers that might face them in practicing research. Previous research was conducted in the Arab region to evaluate the level of knowledge, attitudes and practices in medical students [12–15], however, most of the studies targeted single institutions or universities. Moreover, most of the medical institutions of the region are under-studied, leading to lack of data about the topic. Therefore, in this study, we aim to assess the knowledge, attitudes, practices and perceived barriers of the undergraduate medical students towards research in a wider scale (six countries and 57 universities) than that examined in the present literature. We aim to provide data on the current situation to help develop recommendations and a strategy to improve the medical research field in the Arab world.

## Methods

### Study design and aim

This is a multi-center cross sectional study that aimed primarily to describe the knowledge, attitudes, practices and perceived barriers towards research among undergraduates in medical schools in six Arab countries (Egypt, Algeria, Sudan, Jordan, Syria and Palestine). Then as a secondary objective, associate the described factors with the students' demographics. The current study was conducted in accordance with the STROBE statement for cross sectional studies.

### Setting

The study was conducted via an online questionnaire distributed through the online educational and social media platforms of the participating medical schools. The participating medical schools were selected on basis of feasibility in collecting data from them. See a list of the included medical schools in Additional file 2.

### Participants

All undergraduate medical students in the participating medical schools in the six included countries, who exceeded the age of 18, were eligible and invited to participate in the study by filling-in the questionnaire. All graduates, students in their internship year(s) and Para-medical students were not eligible for participation.

### Instruments used to measure the variables of interest in the study

We used a five-part questionnaire to assess the study variables as follows.

1. First part: included questions to assess the demographic characteristics of the participating students which were Age, gender, residence (Rural/Urban), college, University type (private/governmental), educational level of the parents (below college/college or above), country, academic stage (basic/clinical) and University name (was used to classify the participating universities according to whether or not they rank in the top 1000 universities according to Times Higher Education World University Rankings 2020).
2. Second part (knowledge regarding research assessment questionnaire): aimed to assess the knowledge of medical students regarding basic research information by eight questions. The questions simulated a test with multiple answers and an "I don't know" choice. The questions were reproduced from Memarpour and colleagues [16] the Cronbach's alpha of the scale in the original paper was (0.71) and in the current sample was (0.63).
3. Third part (Attitudes towards research assessment scale): aimed to measure students’ attitudes towards research and consisted of 31 questions answered by three-likert scale, Agree / uncertain /disagree. The scale further sub-divides into five factors, Research usefulness to the profession (nine questions), Research Anxiety (eight questions), Positive attitudes towards research (seven questions), Relevance to life (four questions) and research difficulty (three questions). The Scale was reproduced from "the attitudes towards research scale" [17] Cronbach's alpha in the original paper for the sub-scales ranged from 0.71 to 0.92 and in the current sample ranged from 0.66 to 0.85.
4. Fourth part (practices regarding research assessment questions): Aimed to assess students' Practice towards medical research by eight questions reproduced from Alghamdi et al. [18] The questions included two yes/No questions asking the students whether or not they participated in a research learning workshops before and whether or not they participated in research projects. Then four questions asking about the number of research projects they participated in, the number of publications, the number of research related oral presentations and posters. Then two questions asking about the type of research they participated in and the process of it they were involved in.
5. Fifth part (Barriers towards research practice measurement scale): Aimed to assess perceived barriers towards medical research practice measured by 32 sentences stating certain anticipated barriers reproduced from Memarpour et al. [16] the answer is by three-likert scale, Agree / uncertain /disagree. The Cronbach's alpha in the original paper was (0.88) and in the current sample was (0.9).

The full version of the questionnaire was checked for relevance, Comprehensiveness, face and content validity before the beginning of data collection. Pilot testing was also done among a group of students to ensure clarity of the content of the questionnaire. A full version of the questionnaire can be found in Additional file 3. The questionnaire was distributed through the online platforms in English language for all countries.

### Sampling and sample size calculation

Convenience sampling method was used to acquire the responses from the participants via online distribution of the survey. An independent sample size was calculated for medical students in each one of the included countries via The following equation *n* = z^2^P(1-P)/d^2^[19]. Under a 95% CI, 50% response distribution and 0.05 margin of error, a sample of 384 participants can be considered as a minimal sample to represent the population of medical students in each country.

### Data collection and handling

Allocated teams to each country started the data collection in May 2020 till July 2020. The online questionnaires were distributed through the online platforms of the participating medical schools. Central inspection of the collected data was done to avoid the over-representation of one demographic group over the other.

### Statistical analysis

1. For the knowledge assessment section, scoring of the questions was done by giving the right answer a score of one and the wrong or "I don't know" answer a score of zero yielding a score range of zero to eight for each participant. We adopted a cutoff point for the score as follows: less than 50% as (poor), 50 to 75% as (fair) and over 75% as satisfactory. Description of the knowledge level was done by calculating mean and SD and the frequency of each knowledge level category.
2. For the attitudes assessment section, the answers were given a score of one, two or three for the Agree/uncertain/disagree or the reverse, giving a total score ranging from 3–27 for the research usefulness to the profession, 3–24 for research anxiety, 3–21 for positive attitudes towards research, 3–12 for relevance to life and 3–9 for research difficulty. The higher the score, the higher the factor represented by the score. Description was done by calculation of Mean and SD for each factor.
3. For the practices assessment scale, frequency and percentage was calculated for each of the questions, and the number of research projects, publications, oral presentations and posters was reported as mean number.
4. For the barriers assessment scale, description was done by calculation of Frequency and percentage of students who agreed to each barrier, further more scoring and quantification of the scale was done by giving the answers (disagree/uncertain/Agree) scores of one, two and three yielding a score for each student ranging from 32 to 96. The score was considered as quantification for "burden of barriers" among students, description of the score was done by calculation of Mean and SD and frequency and percentage of the students who scored less than 50%, 50–75%, and more than 75% of the maximum attainable score.

For testing the association between the scores and the demographics, normality of the distribution of the scores was done using the Shapiro–wilk test and accordingly, Mann whitney and Kruskal–wallis tests were used to test the association between the knowledge, attitudes, and barriers scores and the demographics. Furthermore, chi-square test was used to test the difference in frequency of participation in research projects and enrolling to workshops according to the demographics. A P value was considered significant at 0.05. All Analysis was done using IBM SPSS version 24.

## Results

A total number of 2989 undergraduate medical students comprised the study sample, forty percent of them were males and 51.5% were in their clinical years. The study sample was collected from six Arab countries, Egyptian medical students comprised about one quarter of the sample (25.7%), while 17.6%, 16.5%, 14.5%, 14.3% and 11.5% were from Syria, Algeria, Jordan, Palestine and Sudan respectively. Full demographic data is described in Table 1.

### Knowledge regarding research basics among the students

Out of a total score of eight, the mean knowledge score for the whole sample was 2.1 (SD = 1.59) reflecting a generally poor level of knowledge.

Categorization of the students' scores showed that 91.6% of the sample had poor level of knowledge, 8.1% had fair level of knowledge and only nine students (0.3%) had a satisfactory knowledge level (more than 75% of the score).

### Attitudes of the students towards research

The students in the study scored a mean of 24.4 (SD = 2.9) out of 27 in the Factor of "research usefulness for profession", 17.6 (SD = 3.4) out of 24 in the factor regarding "Positive attitudes towards research", 9.2 (SD = 1.8) out of 12 in the factor measuring relevance of research to life, indicating a high level of perceived research usefulness and relevance and a high level of generally positive attitudes towards research.

Also, the students scored a mean score of 15 (SD = 3.9) out of 24 in the factor measuring research Anxiety, and 5.6 (SD = 1.5) out of 9 in the factor measuring research difficulty indicating a moderate level of perceived difficulty and anxiety towards research.

### Practices of students in the field of research

Out of the total study sample, 37.3% attended a research methodology workshops or training, while 33.7% (*n* = 1006) participated in an actual research project.

The mean number of research projects among the whole sample was 1.12 research projects per student, and among those who actually participated in a research project was 2.78 per student. The mean number of publications for the whole sample was 0.2 papers per student and for those who participated in a research project before was 0.5 papers per student. The mean number of scientific posters was 0.5 (SD = 3.4) and 0.75 (SD = 3.1) for oral presentations.

Among the 1006 students who actually participated in a research project, the most common research process that the students participated in was constructing the concept of a research project (46%), followed by doing the literature review (35.5%), Doing data entry (32.2%), writing the research proposal (31.3%), performing data analysis (24.2%), and finally, writing the final manuscript (20.1%).

Among the same 1006 students who participated in research projects, the most common type of studies they participated in was cross-sectional studies (38.6%), followed by case reports (23.9%), systematic reviews and meta-analysis (22.2%), then retrospective clinical trials (15.1%), lab research (13.7%), Randomized controlled trials (RCTS) (12.6%), then finally prospective clinical studies (8.7%). In Egypt, Jordan, Palestine and Sudan, the most common types of research studies among the participants was cross-sectional studies, while in Algeria and Syria was Case reports. Systematic reviews and meta-analysis came second in Egypt and third in Jordan and Syria. See Additional file 1.

### Perceived barriers towards research practice:

The top ten perceived barriers towards research practice in the entire sample were lack of access to lab equipment for lab research (68.1%), priority of education over research (66.8%), lack of time because of educational tasks (66.1%)**,** generally poor attention given to researchers (64.6%), lack of fund (62%), poor collaboration between different academic departments and research centers (61.3%), Insufficient research skills (60.8%), lack of suitable research space (59.9%), lack of professor input (59.2%) and lack of familiarity with research studies (57.8%). The barriers regarding lack of research skills as finding good ideas, writing, statistical analysis, and article submission all were agreed on by more than 50% of the sample. For full details regarding the whole barriers and details for each country see Additional file 1.

Scoring and categorization of the perceived burden of barriers showed that 42.9% of the sample scored (more than 75%) of the maximum attainable score, 43% scored in the (50–75%) range and only 14.1% scored less than 50% of the score, reflecting a generally high burden of perceived barriers towards research among the study participants. The mean score for burden of barriers for the whole sample was 77.8 (SD = 11.00).

### Relationship between knowledge level of the students and their demographics

Being a student in the top1000 Universities, having a father or a mother with a college degree or above and being a student in the clinical years were significantly associated with higher levels of knowledge. Tables 2 and 3.

**Table 1:** Socio-demographic characteristics (*n*= 2989)

Basic characteristics	^a^*n* (%)	Mean (SD)
**Age**		21.3 (2.24)
** Gender**		
Male	1196 (40)	
Female	1793 (60)	
** Country**		
Algeria	494 (16.5)	
Egypt	767 (25.7)	
Jordan	433 (14.5)	
Palestine	427 (14.3)	
Sudan	343 (11.5)	
Syria	525 (17.6)	
** Father education**^a^		
College or above	2145 (72.8)	
Below college	800 (27.2)	
** Mother education**^a^		
College or above	1885 (63.7)	
Below college	1074 (36.3)	
** Residency**		
Rural	854 (28.6)	
Urban	2135 (71.4)	
** University type**		
Private	635 (21.2)	
Public	2354 (78.8)	
** Academic stage**		
** Clinical years**	**1538 (51.5)**	
** Basic years**	**1451 (48.5)**	
** Top1000 Universities (According to Times Higher Education World University Rankings 2020)**		
** Yes (9 universities)**	**523 (17.5%)**	
** No (48 universities)**	**2466 (82.5%)**	

^a^Data for father and mother education is available for 2945 and 2959 participants only.

**Table 2:** Comparisons between knowledge, attitudes, and barriers scores according to Gender, University rank and University type by Mann–whitney test

Scores	Gender		*P*-value	Top1000 Universities		*P*-value	University type		*P*-value
	Male	Female		Yes	No		Public	Private	
Knowledge score	2.08 (1.6)	2.11 (1.5)	0.22	2.47 (1.61	2.02 (1.58)	< 0.001*	2.11 (1.58)	2.05 (1.64)	0.266
Research usefulness for profession	24.0 (3.1)	24.3 (2.7)	0.01*	24.93 (2.64)	24.29 (2.96)	< 0.001*	24.66 (2.69)	23.48 (3.47)	< 0.001*
Research anxiety	15.42 (3.6)	14.71 (4.0)	< 0.001*	14.76 (4.04)	15.05 (3.87)	0.127	15.07 (3.95)	14.71 (3.72)	0.541
Positive attitudes towards research	17.5 (3.3)	17.58 (3.4)	0.69	17.43 (3.65)	17.58 (3.32)	0.886	17.7 (3.3)	16.9 (3.58)	0.004*
Relevance to life	9.03 (1.7)	9.22 (1.8)	< 0.001*	9.33 (1.88)	9.1 (1.79)	0.004*	9.18 (1.82)	9.01 (1.76)	0.009*
Research difficulty	5.70 (1.5)	5.49 (1.6)	0.75	5.69 (1.62)	5.55 (1.52)	0.081	5.55 (1.55)	5.68 (1.5)	0.487
Burden of Barriers score	77.5 (11)	77.97 (10.9)	0.366	77.94 (10.35)	77.3 (11.1)	0.669	78.14 (11.01)	76.43 (10.72)	0.001*

Scores are presented as Mean (SD)

**Table 3:** Comparisons between knowledge, attitude, and barriers scores according to Father and mother education, residence and Academic stage by Mann–whitney test

hba	Father education		*P*-value	Mother education		*P*-value	Residence		*P*-value	Academic stage		*P*-value
	Below college	Above college		Below college	Above college		Rural	Urban		Basic	Clinical	
Knowledge score	1.89 (1.57)	2.18 (1.6)	< 0.001*	1.88 (1.5)	2.22 (1.63)	< 0.001*	2.01 (1.59)	2.13 (1.59)	0.07	1.84 (1.52)	2.34 (1.62)	< 0.001*
Research usefulness for profession	23.55 (3.32)	24.72 (2.68)	< 0.001*	23.53 (3.32)	24.9 (2.53)	< 0.001*	23.97 (3.15)	24.58 (2.8)	< 0.001*	24.26 (3.01)	24.54 (2.82)	0.15
Research anxiety	15.34 (3.63	14.86 (3.99)	< 0.001*	15.22 (3.66)	14.87 (4.03)	< 0.001*	15.28 (3.74)	14.88 (3.96)	0.002*	15.28 (3.84)	14.73 (3.94)	< 0.001*
Positive attitudes towards research	17.13 (3.27)	17.71 (3.4)	< 0.001*	17.06 (3.35)	17.83 (3.37)	< 0.001*	17.39 (3.44)	17.62 (3.36)	0.94	17.7 (3.33)	17.4 (3.43)	< 0.001*
Relevance to life	9.04 (1.74)	9.18 (1.23)	< 0.001*	9.01 (1.79)	9.22 (1.82)	< 0.001*	8.94 (1.78)	9.23 (1.82)	< 0.001*	9.06 (1.82)	9.22 (1.8)	0.07
Research difficulty	5.64 (1.42)	5.55 (1.58)	< 0.001*	5.6 (1.46)	5.56 (1.58)	0.01*	5.52 (1.47)	5.6 (1.57)	0.001*	5.62 (1.54)	5.53 (1.54)	0.16
Burden of Barriers score	76.21 (11.24)	78.36 (10.82)	< 0.001*	76.69 (11.04)	78.39 (10.9)	0.001*	76.86 (11.4)	78.14 (10.78)	0.023*	76.6 (11.31)	78.89 (10.53)	< 0.001*

Scores are presented as Mean (SD)

Students in Jordan and Palestine had the highest level of knowledge, followed by students in Algeria, Egypt and Syria and the least score was in Sudanese students. The differences were statistically significant. Table 4.

**Table 4:** Comparisons between knowledge, attitude, and barriers scores according to the country of the student by Kruskal Wallis test

	Country						*P*-value
	Algeria	Egypt	Jordan	Palestine	Sudan	Syria	
Knowledge score	2.15 (1.39)	2.11 (1.60	2.19 (1.66)	2.18 (1.64)	1.77 (1.48)	2.11 (1.70)	0.005*
Research usefulness for profession	24.99 (2.34)	25 (2.18)	25.16 (2.61)	24.45 (2.84)	24.33 (2.81)	22.42 (3.68)	< 0.001*
Research anxiety	15.7 (3.75)	14.96 (3.95)	14.53 (4.16)	14.69 (4.12)	14 (6.99)	15.69 (3.26)	< 0.001*
Positive attitudes towards research	18.17 (2.37)	17.7 (3.48)	18.24 (3.41)	13.58 (3.5)	17.27 (3.85)	16.37 (3.2)	< 0.001*
Relevance to life	9.43 (1.8)	9.22 (1.9)	9.23 (1.74)	9.24 (1.89)	9 (1.85)	8.74 (1.53)	< 0.001*
Research difficulty	5.5 (1.48)	5.44 (1.54)	5.45 (1.47)	5.36 (1.54)	5.4 (2.67)	5.95 (1.23)	0.128
Burden of Barriers score	77.97 (9.8)	77.76 (10.94	81.32 (11.36	75 (10.5)	78.35 (11.43)	76.43 (10.9)	< 0.001*

Scores presented as Mean (SD)

### Relationship between attitudes of the students and their demographics

Female gender was associated with higher perception of research usefulness to the profession and relevance to life, while males had significantly higher scores regarding research anxiety. Tables 2 and 3.

Being a student in one of the top 1000 universities was associated with higher scores regarding research usefulness to the profession and relevance to life. Tables 2 and 3.

Being a student in a governmental (public) university was significantly associated with more general positive attitude towards research and higher scores regarding research usefulness and relevance. Tables 2 and 3.

Having a father or a mother with a university degree or above was significantly associated with more positive attitudes towards research, higher perception of its usefulness and relevance, and lower scores regarding research Anxiety and difficulty. Tables 2 and 3.

Living in urban area was associated with higher perception of research usefulness and relevance and less perception of research anxiety. Tables 2 and 3.

Being a student in the basic years was associated with more positive attitudes towards research and yet higher anxiety towards its practice. Tables 2 and 3.

### Relationship between practices of students and their demographics

Being a student in one of the top 1000 universities, being in a private university, being in the clinical years of medical school and having a father or a mother with a university degree or above was associated with higher frequency of participation in research projects and enrollment in research workshops. Tables 5 and 6.

**Table 5:** Chi-Square analysis for comparison of the Frequency of participation in research projects according to the students demographics

	Yes (*n* = 1006)	No (*n* = 1983)	*P*-value
Gender			
Female	618(34.5%)	1175(65.5%)	0.251
male	388(32.4%)	808(6706%)	
Uni_TOP1000			
No	760(30.8%)	1706(69.2%)	< 0.001*
Yes	246(47.0%)	277(53.0%)	
University type			
Private	248(39.2%)	385(60.8%)	0.001*
Public	757 (32.2%)	1597(67.8%)	
Father education			
Below college	224(28.0%)	576(72.0%)	< 0.001*
College and above	776(36.2%)	1369(63.8%)	
Mother education			
Below college	306(28.5%)	768(71.5%)	< 0.001*
College and above	695(36.9%)	1190(63.1%)	
Residence			
Rural	277(32.4%)	577(67.6%)	0.372
urban	729(34.1%)	1406(65.9%)	
Academic stage			
Basic	416 (28.7%)	1035 (71.3%)	< 0.001*
Clinical	590 (38.4%)	948 (61.6%)	
Country			
Algeria	88 (17.8%)	406 (82.2%)	.32
Egypt	325 (42.4%)	422 (57.6%)	
Jordan	157 (36.3%)	276 (63.7%)	
Palestine	202 (47.3%)	225 (52.7%)	
Sudan	134 (39.1%)	209 (60.9%)	
Syria	100 (19%)	425 (81%)

**Table 6:** Chi-Square analysis for comparison of the Frequency of enrollment in research learning workshops according to the students' demographics

	Yes (*n* = 1115)	No (*n* = 1874)	*P*-value
Gender			
Female	652(36.4%)	1141 (63.6%)	.2
male	463(38.7%)	733 (61.3%)	
Uni_TOP1000			
No	880(35.7%)	1586 (64.3%)	< 0.001*
Yes	235(44.9%)	288 (55.1%)	
University type			
Private	268(42.2%)	367 (57.8%)	.005*
Public	847(36.0%)	1507 (64%)	
Father education			
Below college	266(33.3%)	534 (66.7%)	.004*
College and above	839(39%)	1306 (61%)	
Mother education			
Below college	358(33.3%)	716 (66.7%)	.001*
College and above	749(39.7%)	1136 (60.3%)	
Residence			
Rural	329(38.5%)	525 (61.5%)	.4
Urban	786(36.8%)	1349 (63.2%)	
Academic stage			
Basic	476 (32.8%)	975 (67.2%)	< 0.001*
Clinical	639 (41.5%)	899 (58.5%)	
Country			
Algeria	91 (18.4%)	403 (81.6%)	.16
Egypt	367 (47.8%)	400 (52.2%)	
Jordan	149 (34.4%)	284 (65.6%)	
Palestine	212 (49.6%)	215 (50.4%)	
Sudan	138 (40.2%)	205 (59.8%)	
Syria	158 (30.1%)	367 (69.9%)	

### Relationship between perceived burden of barriers by the students and their demographics

Being in a governmental (public) university, having a father or a mother with a college or above degree, living in urban places and being in the clinical years of medical education was associated with higher perceived level of barriers indicated by the burden of barriers score. Tables 2 and 3

Students studying in Jordan had the highest burden of barriers score, followed by Students from Sudan, Algeria, and Egypt with minimal differences, then Syrian students, and the least perceived level of barriers was found in students studying in Palestine. The differences were statistically significant. Table 4.

## Discussion

Our results showed that the sample of this study had poor knowledge levels, and yet, a high level of perception of research as relevant and useful practice with moderate anxiety towards it. The perceived burden of barriers was generally high, and presented as either lack of time and resources or lack in the research knowledge and needed skills. One third of the students participated in research projects, with low publication rates in contrast with the participation rate. The study mainly points out the un-translated potential of the students which lies in their positive perception and attitudes towards research, which does not translate into actual knowledge or practice.

Our results are compatible with results from other studies conducted in countries with the same educational situation; low knowledge level with high positive attitudes towards research was seen in Egypt and the Gulf countries [14, 20, 21]**.** Studies from Jordan and Syria reported the same gap in knowledge despite the presence of positive attitudes [12, 13]**.** Moderate level of knowledge with less positive attitudes was however seen in one Egyptian study [15]**.** Regarding the situation in Eastern non-Arab countries, Studies from Iran, Pakistan and India reported more adequate levels of knowledge, with high levels of positive attitudes [16, 22, 23]**.** Studies from countries with better medical education and more institutional student research programs in Europe and the United States show a growing interest in research among the undergraduates [24, 25], positive feelings towards research, and more motivation and optimism to pursue careers in research with a higher level of confidence about the research skills specially after research electives or training programs [26–28].

Regarding the barriers encountered by the students trying to practice research, the un-availability of sufficient time because of the overwhelming educational tasks was a prevalent barrier in our study and was also reported by other studies [18, 23]. Integration of research projects as part of the curricular requirements can help provide enough time and attention to them. The lack of the knowledge and the skills needed to perform research in addition to the lack of mentoring is a prevalent barrier in our study and others [14, 20, 29–31]. A proposed solution can be the development of learning programs that focus on improving the students' research skills under the supervision of the faculty staff members.

The students in the current study had a low number of publications compared to the number of research projects they participated in (2.8 projects VS 0.5 publications per student). Although there are no studies that report the number of student-authored publications in the Arab region [32], an increase in the number of student and trainee authored publications is generally noted (up to 14.5% of all publications) [33]. A 10% of the research output of the top ten universities in the world is co-authored by undergraduates [34].

However, the low number of publications compared to the number of research projects in this current study can be possibly explained by the above reported barriers. The lack of proper mentoring and sufficient research skills may lead the research projects to not reach the point of peer review and publication. Another possible explanation is that some of the undergraduate research projects are mainly educational with little chance of its output to be publishable. Although many students may have the particular goal of publishing research to enhance their starting careers, the mere participation or exposure to research in the undergraduate level seems to increase and facilitate research productivity after graduation [35]. The most prevalent types of research that the students participated in were cross-sectional studies, case reports and reviews, which require less clinical and research skills and mentoring than other clinical studies. While the potential of the students can be directed towards more feasible types of research as a start, this should not obviate the need for proper mentoring and training and it should be noted that these types of research may contribute to their lower chances in publication, as narrative reviews and case reports might be harder to publish.

The current study shows a gender difference in the perception of research, as females showed higher levels of perception of its relevance and usefulness with males showing higher anxiety towards it. However, no difference was noted in the levels of knowledge, participation in research workshops or in research projects complying with previous literature [14, 36]. Students in their clinical years showed better levels of knowledge, practice and perception of research indicating that students upgrade during the medical school, previous literature supports this finding [14, 15, 20, 23]. Higher standards of living as living in urban areas and having parents with higher education was associated with better knowledge, attitudes and practices in this study sample.

To our knowledge, this is the first study to assess the knowledge, attitudes, practices and barriers on a wide scale (6 countries, 57 Universities). The high number of recruited participants, and the fulfillment of the minimum sample size in each country in addition to the balance between the representation of basic and clinical years supports the generalizability of the results. However, the use of convenience sampling and online surveying of students stands as a limitation that might have led to an over-estimation of the variables of knowledge, positive attitudes and practices, as students who have a particular interest in research are more likely to participate and take the survey. Although the data from this study can be adequately generalizable to most of the Arab countries with the same educational background, it lacks representativeness to some Arab regions as the gulf area.

### Implication of findings

It is immediately clear that the situation of students' knowledge and practices in the research field needs improvement. The cultivation of the students' positive attitudes towards research and their perception of its relevance and usefulness should be the starting point in discussing new strategies to include the students in the research field and use their potential.

The most prevalent barriers lied in the presence of overwhelming educational tasks that leave no place for research involvement in addition to absence of proper mentoring and guidance. Integration of research as an educational task can be a proposed solution to these both problems. Also, the development of structured research skills learning programs with practical evidence of its affectivity can improve the students' skills and increase their productivity.

## Conclusion

Medical students in the included countries showed low levels of knowledge despite having positive attitudes towards research. The main encountered barriers towards practice were the lack of time, the lack of access to resources, and the lack of skills and mentoring. One third of the students practiced research, with a low number of publications compared to the number of research projects they participated in. in general, the students' positive attitudes do not translate into actual knowledge and practice. Proper integration of research into curricular activities with providing proper mentoring from the educational staff can help reduce the barriers faced by the students and increase their productivity.

## Supplementary Information

**Additional file 1.** Includes further information about the most frequent research type and the frequency of the encountered barriers.

**Additional file 2.** Includes the names of the included universities.

**Additional file 3.** Includes the questionnaire.

## Data Availability

The datasets used and/or analyzed during the current study are available from the corresponding author on reasonable request.
