# Supplementary material for: Knowledge, attitudes, practices and perceived barriers towards research in undergraduate medical students of six Arab countries
Source: BMC Med Educ. 2022 Jan 18;22:44. doi: 10.1186/s12909-022-03121-3 (PMC8767733; doi:10.1186/s12909-022-03121-3)
Supplement: Supplementary file 1 — Additional file 1. Includes further information about the most frequent research type and the frequency of the encountered barriers. [file 12909_2022_3121_MOESM1_ESM.doc]

**Supplementary material (Additional file 1)**

Table 1: three most common research types practiced by students in each country

| Country | Most common research type (top 3) |
| --- | --- |
| Algeria | Case reports (27.3%), Retrospective clinical trials (22.7%), and lab research (21.6%) |
| Egypt | Cross-sectional studies (37.5%), Review articles (32.6%), Case reports (15.1%) |
| Jordan | Cross-sectional (37.6%), Retrospective clinical trials (25.5%), Review articles (24.8%) |
| Palestine | Cross-sectional (42.1%), Case reports (29.2%), Retrospective clinical trials (17.3%) |
| Sudan | Cross-sectional (52.2%), Case reports (17.9%), lab research (14.9%) |
| Syria | Case reports (50%), Cross sectional (41%), Review articles (19%) |

Table 2: Percentage of agreement to each barrier in the whole sample

| Barrier number | Percent of people whom agreed to face this barrier (ALL) |
| --- | --- |
| Lack of access to laboratory equipment for performing research project | 68.1 |
| Priority on education over research in university | 66.8 |
| Lack of time to do research because of educational tasks | 66.1 |
| Poor attention given to researchers and creative faculty | 64.6 |
| Lack of timely funding of research & lack of funds | 62 |
| Poor collaboration between departments and research centers | 61.3 |
| Insufficient research skills | 60.8 |
| Lack of suitable research space | 59.9 |
| Lack of professor input with students | 59.2 |
| Lack of familiarity with research studies | 57.8 |
| Lack of familiarity with statistical analysis | 57.5 |
| lack of cooperation between research centers | 57 |
| Lack of access to studies across the country | 55.9 |
| Lack of skills for writing papers | 54.5 |
| Personal economic & financial problems | 54.4 |
| Lack of ability to publish article after the completion of the project | 54.2 |
| Lack of research needs and priorities in university health system | 54.2 |
| Lack of significant income to do research | 53.1 |
| Dissatisfaction with encouragement of researchers to do research | 53 |
| Lack of good research ideas | 52.6 |
| Boring and difficult to research because of the lack of skill | 52.6 |
| Lack of appropriate databases | 51.5 |
| Lack of research applications in personal life and professional job | 51.5 |
| Lack of coordination of research priorities with research ideas | 49.4 |
| Inappropriate or insufficient guidance for writing | 49.4 |
| Lack of familiarity with research proposal writing | 48.2 |
| Lack of confidence in potential for completing research | 47.5 |
| Prefer to use the free time to do other task | 46.6 |
| Inappropriate or insufficient consultation before drafting research proposals | 44.7 |
| Fear of making mistakes in research and being blamed by others | 43.6 |
| Lack of interest in research | 40.5 |
| Lack of skills for submitting articles | 37.4 |

Tables 3,4,5,6,7,8 Percentage of agreement to each barrier in each country

| Barrier number | Percent of people whom agreed to face this barrier (Algeria) |
| --- | --- |
| Lack of access to laboratory equipment for performing research project | 82.4 |
| Priority on education over research in university | 79.4 |
| Poor collaboration between departments and research centers | 77.3 |
| Poor attention given to researchers and creative faculty | 76.1 |
| Lack of suitable research space | 75.5 |
| Lack of timely funding of research & lack of funds | 74.1 |
| Dissatisfaction with encouragement of researchers to do research | 70.6 |
| Lack of access to studies across the country | 70.4 |
| Lack of professor input with students | 70.2 |
| lack of cooperation between research centers | 68.8 |
| Lack of appropriate databases | 65.2 |
| Lack of research applications in personal life and professional job | 61.7 |
| Personal economic & financial problems | 61.5 |
| Lack of familiarity with research studies | 59.9 |
| Lack of time to do research because of educational tasks | 59.7 |
| Lack of coordination of research priorities with research ideas | 58.9 |
| Lack of familiarity with research proposal writing | 56.9 |
| Lack of familiarity with statistical analysis | 56.7 |
| Insufficient research skills | 54.3 |
| Lack of skills for writing papers | 48.8 |
| Lack of interest in research | 48 |
| Lack of skills for submitting articles | 47.8 |
| Lack of confidence in potential for completing research | 46.6 |
| Inappropriate or insufficient consultation before drafting research proposals | 44.1 |
| Boring and difficult to research because of the lack of skill | 43.3 |
| Lack of research needs and priorities in university health system | 43.3 |
| Prefer to use the free time to do other task | 43.3 |
| Lack of significant income to do research | 42.5 |
| Inappropriate or insufficient guidance for writing | 42.3 |
| Fear of making mistakes in research and being blamed by others | 41.5 |
| Lack of ability to publish article after the completion of the project | 40.3 |
| Lack of good research ideas | 26.9 |

| Barrier number | Percent of people whom agreed to face this barrier (Egypt) |
| --- | --- |
| Lack of time to do research because of educational tasks | 71.8 |
| Priority on education over research in university | 68.1 |
| Lack of access to laboratory equipment for performing research project | 67.5 |
| Poor attention given to researchers and creative faculty | 65.7 |
| Insufficient research skills | 65.1 |
| Poor collaboration between departments and research centers | 62.1 |
| Lack of professor input with students | 60.6 |
| Lack of research needs and priorities in university health system | 60.5 |
| Lack of skills for writing papers | 60.1 |
| Lack of timely funding of research & lack of funds | 59.2 |
| Lack of familiarity with statistical analysis | 59.1 |
| Lack of skills for submitting articles | 58.8 |
| Lack of familiarity with research studies | 58.4 |
| Lack of suitable research space | 58.1 |
| Lack of significant income to do research | 56.6 |
| Boring and difficult to research because of the lack of skill | 56.5 |
| Lack of familiarity with research proposal writing | 55.7 |
| Personal economic & financial problems | 54.6 |
| lack of cooperation between research centers | 54.1 |
| Dissatisfaction with encouragement of researchers to do research | 51.9 |
| Lack of coordination of research priorities with research ideas | 51.9 |
| Lack of confidence in potential for completing research | 51.6 |
| Lack of ability to publish article after the completion of the project | 51 |
| Lack of access to studies across the country | 50.6 |
| Inappropriate or insufficient guidance for writing | 50.5 |
| Prefer to use the free time to do other task | 49 |
| Lack of research applications in personal life and professional job | 48.9 |
| Lack of appropriate databases | 46 |
| Inappropriate or insufficient consultation before drafting research proposals | 43.5 |
| Fear of making mistakes in research and being blamed by others | 43.3 |
| Lack of interest in research | 40.7 |
| Lack of good research ideas | 39.5 |

| Barrier number | Percent of people whom agreed to face this barrier (Jordan) |
| --- | --- |
| Priority on education over research in university | 77.6 |
| Lack of time to do research because of educational tasks | 76.4 |
| Lack of timely funding of research & lack of funds | 72.1 |
| Lack of access to laboratory equipment for performing research project | 71.8 |
| Poor attention given to researchers and creative faculty | 70.4 |
| Insufficient research skills | 69.5 |
| Lack of familiarity with statistical analysis | 69.3 |
| Lack of familiarity with research studies | 68.8 |
| Lack of skills for submitting articles | 68.8 |
| Poor collaboration between departments and research centers | 67.7 |
| Lack of skills for writing papers | 67.2 |
| Boring and difficult to research because of the lack of skill | 66.1 |
| Lack of familiarity with research proposal writing | 65.6 |
| Lack of suitable research space | 65.4 |
| lack of cooperation between research centers | 65.1 |
| Lack of research needs and priorities in university health system | 64.4 |
| Inappropriate or insufficient guidance for writing | 63 |
| Lack of professor input with students | 63 |
| Lack of significant income to do research | 62.8 |
| Lack of coordination of research priorities with research ideas | 62.4 |
| Lack of appropriate databases | 61.7 |
| Lack of ability to publish article after the completion of the project | 61.7 |
| Lack of access to studies across the country | 60.3 |
| Dissatisfaction with encouragement of researchers to do research | 58.9 |
| Inappropriate or insufficient consultation before drafting research proposals | 58.2 |
| Personal economic & financial problems | 57 |
| Lack of confidence in potential for completing research | 56.8 |
| Prefer to use the free time to do other task | 56.4 |
| Lack of research applications in personal life and professional job | 53.3 |
| Fear of making mistakes in research and being blamed by others | 52 |
| Lack of good research ideas | 46.2 |
| Lack of interest in research | 40.9 |

| Barrier number | Percent of people whom agreed to face this barrier (Palestine) |
| --- | --- |
| Lack of time to do research because of educational tasks | 63 |
| Insufficient research skills | 58.8 |
| Priority on education over research in university | 58.1 |
| Lack of access to laboratory equipment for performing research project | 57.4 |
| Lack of timely funding of research & lack of funds | 55 |
| Lack of skills for writing papers | 53.6 |
| Lack of skills for submitting articles | 51.3 |
| Poor attention given to researchers and creative faculty | 50.8 |
| Lack of suitable research space | 50.1 |
| Lack of familiarity with research studies | 49.9 |
| Lack of professor input with students | 48.2 |
| Poor collaboration between departments and research centers | 47.8 |
| Lack of familiarity with statistical analysis | 47.5 |
| Lack of familiarity with research proposal writing | 47.1 |
| Boring and difficult to research because of the lack of skill | 46.8 |
| Lack of significant income to do research | 45.9 |
| Lack of research needs and priorities in university health system | 45.4 |
| lack of cooperation between research centers | 45.2 |
| Lack of research applications in personal life and professional job | 45 |
| Lack of access to studies across the country | 44.5 |
| Inappropriate or insufficient guidance for writing | 44 |
| Prefer to use the free time to do other task | 43.1 |
| Lack of ability to publish article after the completion of the project | 42.4 |
| Personal economic & financial problems | 42.2 |
| Lack of appropriate databases | 41 |
| Lack of confidence in potential for completing research | 41 |
| Dissatisfaction with encouragement of researchers to do research | 40.3 |
| Fear of making mistakes in research and being blamed by others | 37.7 |
| Lack of coordination of research priorities with research ideas | 37 |
| Inappropriate or insufficient consultation before drafting research proposals | 36.5 |
| Lack of good research ideas | 35.8 |
| Lack of interest in research | 30 |

| Barrier number | Percent of people whom agreed to face this barrier (Sudan) |
| --- | --- |
| Insufficient research skills | 70.8 |
| Lack of access to laboratory equipment for performing research project | 70 |
| Lack of time to do research because of educational tasks | 68.5 |
| Lack of timely funding of research & lack of funds | 67.9 |
| Lack of significant income to do research | 64.7 |
| Poor attention given to researchers and creative faculty | 64.4 |
| Priority on education over research in university | 64.1 |
| Lack of access to studies across the country | 63.8 |
| Personal economic & financial problems | 63 |
| Lack of familiarity with research studies | 63 |
| Lack of skills for writing papers | 63 |
| Lack of familiarity with statistical analysis | 61.2 |
| Lack of skills for submitting articles | 61.2 |
| Poor collaboration between departments and research centers | 60.3 |
| Lack of research needs and priorities in university health system | 60.1 |
| lack of cooperation between research centers | 59.8 |
| Boring and difficult to research because of the lack of skill | 58 |
| Lack of professor input with students | 57.7 |
| Inappropriate or insufficient guidance for writing | 57.1 |
| Lack of suitable research space | 56.9 |
| Lack of familiarity with research proposal writing | 56.6 |
| Lack of appropriate databases | 56 |
| Lack of research applications in personal life and professional job | 55.7 |
| Lack of coordination of research priorities with research ideas | 54.2 |
| Lack of ability to publish article after the completion of the project | 51.6 |
| Inappropriate or insufficient consultation before drafting research proposals | 51.3 |
| Dissatisfaction with encouragement of researchers to do research | 51 |
| Prefer to use the free time to do other task | 49 |
| Lack of confidence in potential for completing research | 46.6 |
| Fear of making mistakes in research and being blamed by others | 46.4 |
| Lack of interest in research | 44 |
| Lack of good research ideas | 42.3 |

| Barrier number | Percent of people whom agreed to face this barrier (Syria) |
| --- | --- |
| Lack of access to laboratory equipment for performing research project | 59.8 |
| Poor attention given to researchers and creative faculty | 58.5 |
| Lack of time to do research because of educational tasks | 56 |
| Lack of professor input with students | 53.3 |
| Lack of suitable research space | 53 |
| Priority on education over research in university | 53 |
| Lack of familiarity with statistical analysis | 52.2 |
| lack of cooperation between research centers | 51.2 |
| Poor collaboration between departments and research centers | 51.2 |
| Lack of access to studies across the country | 50.3 |
| Lack of research needs and priorities in university health system | 50.3 |
| Personal economic & financial problems | 49.5 |
| Lack of familiarity with research studies | 49.1 |
| Insufficient research skills | 48.6 |
| Lack of timely funding of research & lack of funds | 48.4 |
| Lack of skills for submitting articles | 48 |
| Lack of significant income to do research | 48 |
| Lack of skills for writing papers | 47 |
| Lack of research applications in personal life and professional job | 46.9 |
| Lack of familiarity with research proposal writing | 46.1 |
| Boring and difficult to research because of the lack of skill | 45.7 |
| Dissatisfaction with encouragement of researchers to do research | 45 |
| Lack of coordination of research priorities with research ideas | 44.4 |
| Lack of appropriate databases | 44 |
| Lack of ability to publish article after the completion of the project | 43.2 |
| Inappropriate or insufficient guidance for writing | 42.7 |
| Fear of making mistakes in research and being blamed by others | 42.1 |
| Lack of confidence in potential for completing research | 40.6 |
| Prefer to use the free time to do other task | 39.4 |
| Lack of interest in research | 39.2 |
| Inappropriate or insufficient consultation before drafting research proposals | 38.1 |
| Lack of good research ideas | 35 |
