# Supplementary material for: Knowledge, attitudes, practices and perceived barriers towards research in undergraduate medical students of six Arab countries
Source: BMC Med Educ. 2022 Jan 18;22:44. doi: 10.1186/s12909-022-03121-3 (PMC8767733; doi:10.1186/s12909-022-03121-3)
Supplement: Supplementary file 2 — Additional file 2. Includes the names of the included universities. [file 12909_2022_3121_MOESM2_ESM.doc]

| Country | University | Frequency | total |
| --- | --- | --- | --- |
| Algeria (10 Universities) | Université d'Alger | 44 | 494 |
|  | University of Batna | 68 |  |
|  | University of Blida | 33 |  |
|  | University of Bejaia | 59 |  |
|  | University of Constantine | 92 |  |
|  | University of Ouargla | 50 |  |
|  | University of Sidi bel abbes | 32 |  |
|  | University of Tlemcen | 41 |  |
|  | Université d'Oran | 38 |  |
|  | University of Algiers | 37 |  |
| Egypt (18 Universities) | 6 October University | 30 | 767 |
|  | Ain-shams University | 81 |  |
|  | AlAzhar Assuit University | 74 |  |
|  | Al-Azhar cairo university | 40 |  |
|  | Alexandria University | 65 |  |
|  | Assiut university | 42 |  |
|  | Azhar Damietta University | 34 |  |
|  | Beni-suif university | 24 |  |
|  | Cairo University | 48 |  |
|  | Fayoum University | 33 |  |
|  | Masr University for science and tech0logy | 30 |  |
|  | Me0ufia University | 45 |  |
|  | Tanta University | 31 |  |
|  | Zagazig University | 41 |  |
|  | Benha University | 27 |  |
|  | Kafr elsheikh university | 38 |  |
|  | Mansoura University | 35 |  |
|  | South Valley University | 49 |  |
| Jordan (5 Universities) | Jordanian University of science and tech0logy | 122 | 433 |
|  | Jordon University | 82 |  |
|  | Mu’tah University | 58 |  |
|  | The Hashemite University | 111 |  |
|  | Yarmouk University | 60 |  |
| Palestine (5 Universities) | Al-Quds University | 107 | 427 |
|  | An-Najah National University | 87 |  |
|  | Islamic University | 110 |  |
|  | PPU (PALESTINE POLYTECHNIC UNIVERSITY) | 53 |  |
|  | Al-Azhar | 70 |  |
| Sudan (10 Universities) | Alfasher University | 23 | 343 |
|  | University of Khartoum | 51 |  |
|  | Algadarif University | 24 |  |
|  | Alzaim Alazhry | 24 |  |
|  | Bahri University | 82 |  |
|  | Karary University | 46 |  |
|  | Kassala University | 23 |  |
|  | Nile Valley University | 23 |  |
|  | Rea sea university | 23 |  |
|  | University of Gezira | 24 |  |
| Syria (9 universities) | Aleppo University | 68 | 525 |
|  | Damascus University | 79 |  |
|  | Hama University | 55 |  |
|  | Syrian University | 53 |  |
|  | Al andalus University | 53 |  |
|  | Al Baath University | 50 |  |
|  | Tartous University | 56 |  |
|  | Teshreen University | 58 |  |
|  | University of Kalamoon | 53 |  |
| Total (57 Universities) | | 2989 | 2989 |
