# Supplementary material for: Knowledge, attitudes, practices and perceived barriers towards research in undergraduate medical students of six Arab countries
Source: BMC Med Educ. 2022 Jan 18;22:44. doi: 10.1186/s12909-022-03121-3 (PMC8767733; doi:10.1186/s12909-022-03121-3)
Supplement: Supplementary file 3 — Additional file 3. Includes the questionnaire. [file 12909_2022_3121_MOESM3_ESM.doc]

| Dear Colleague,  The following questionnaire is designed to help our researchers to “Evaluate attitude to, knowledge of and barriers toward research among medical science students”. All the data in this questionnaire will be used only for research purposes. No personal data are required. We would be grateful if you would help us by completing the form. By agreeing to complete this form, you are giving us permission to use the data in our research project. | |
| --- | --- |
| **Student demographic information** | |
| 2-Age:…………………………………… | 1-Gender: A) male B)female |
| 4- Type of University you enroll:  A)Governmental B)Private | 3-Country:  A)Algeria B)Egypt C)Jordan D)Palestine E)Sudan F) Syria |
| 6- Mother educational status:  A) Illiterate/ read & write B) Primary/preparatory C) Secondary D) College or higher | 5- Father educational status:  A) Illiterate/ read & write B) Primary/preparatory C) Secondary D) College or higher |
| 5-Academic stage:  A)basic year B)clinical year c) graduate | 5-Residence:  A)Rural B)Urban |
|  | College: (name of college in each country) |

| **Assessment of student knowledge of research**  (this part of the questionnaire aims to assess your knowledge of medical research by asking about some basic concepts, please choose the first answer comes to your mind and feel free to choose “I don’t know” if you don’t. | | | |
| --- | --- | --- | --- |
| **1-A scale from 1 to 5 (like grades on the examination) is called:**  A) Ratio scale B) Ordinal C) Nominal D) Interval E) I don’t Know | | | |
| **2-Which following software is used for reference of science article?**  A)SPSS B)Access C)Concept Map D)End Note E) I don’t Know | | | |
| **3-What is the variable scale of blood type?** A)Nominal B)Relative C)Distance D)Serial E) I don’t Know | | | |
| **4-Which way of writing reference is approved and for writing general medical dissertations and most medical journals?**  A)Vancouver B)Harvard C)None D)Chicago E) I don’t Know | | | |
| **5-What is the definition of Medline?**  A) The first and best known on-line medical journals  B) Association for informed medical providers  C) Print format chosen medical  D) Medical database  E) I don’t know | | | |
| **6-In which part of an article do you talk about the study limitations?**  A) Acknowledgment B) Methods and Materials C) Discussion D) Introduction E) I don’t Know | | | |
| **7-Which of the following types of research sees sample loss more commonly**  A) Clinical trial B) cross Sectional C) Case – control D) Cohort E) I don’t Know | | | |
| **8-Which item is not part of a scientific original paper?**  A) Discussion B) Introduction C) Letters to the Editor D) Methods and Materials  E) I don’t Know | | | |
| **Agree** | **No opinion/ uncertain** | **Disagree** | **Student attitude toward research & assessment approach** |
| This part of the questionnaire aims to assess your attitude towards research as a concept, please choose the answer that best describes what you feel about the sentence. You might feel that some questions are repeated, it is ok. Please answer the whole questions. | | | |
|  |  |  | 1- Research makes me anxious |
|  |  |  | 2- Research should be taught to all students |
|  |  |  | 3- I enjoy research |
|  |  |  | 4- Research is interesting |
|  |  |  | 5- I like research |
|  |  |  | 6- I feel insecure concerning the analysis of research data |
|  |  |  | 7- Research scares me |
|  |  |  | 8- Research is useful for my career |
|  |  |  | 9- I find it difficult to understand the concepts of research |
|  |  |  | 10- I make many mistakes in research |
|  |  |  | 11- I have trouble with arithmetic |
|  |  |  | 12- I love research |
|  |  |  | 13- I am interested in research |
|  |  |  | 14-Research is connected to my field of study |
|  |  |  | 15- Most students benefit from research |
|  |  |  | 16- Research is stressful |
|  |  |  | 17- Research is very valuable |
|  |  |  | 18- Research makes me nervous |
|  |  |  | 19- I use research in my daily life |
|  |  |  | 20- The skills I have acquired in research will be helpful to me in the future |
|  |  |  | 21- Research is useful to every professional |
|  |  |  | 22- Knowledge from research is as useful as writing |
|  |  |  | 23- Research is irrelevant to my life |
|  |  |  | 24- Research should be indispensable in my professional training |
|  |  |  | 25- Research is complicated |
|  |  |  | 26- Research thinking does not apply to my personal life |
|  |  |  | 27- I will employ research approaches in my profession |
|  |  |  | 28- Research is difficult |
|  |  |  | 29- I am inclined to study the details of research procedures carefully |
|  |  |  | 30- Research-orientated thinking plays an important role in my daily life |
|  |  |  | 31- Research is a complex subject |
| **Agree** | **No opinion/ uncertain** | **Disagree** | **Assess the barriers to research students** |
| This part of the questionnaire aims to assess your perception of the barriers that stands against undergraduate research practice, choose the answer that best describes your opinion regarding each sentence, please answer the whole sentences. | | | |
|  |  |  | 1- Lack of timely funding of research & lack of funds |
|  |  |  | 2- Lack of appropriate databases |
|  |  |  | 3- Lack of access to laboratory equipment for performing research project |
|  |  |  | 4- Lack of access to studies across the country |
|  |  |  | 5- lack of cooperation between research centers |
|  |  |  | 6- Lack of suitable research space |
|  |  |  | 7- Dissatisfaction with encouragement of researchers to do research |
|  |  |  | 8- Poor attention given to researchers and creative faculty |
|  |  |  | 9- Priority on education over research in university |
|  |  |  | 10- Lack of time to do research because of educational tasks |
|  |  |  | 11- Poor collaboration between departments and research centers |
|  |  |  | 12- Personal economic & financial problems |
|  |  |  | 13- Insufficient research skills |
|  |  |  | 14- Lack of familiarity with research studies |
|  |  |  | 15- Lack of familiarity with statistical analysis |
|  |  |  | 16- Lack of skills for writing papers |
|  |  |  | 17- Lack of skills for submitting articles |
|  |  |  | 18- Lack of familiarity with research proposal writing |
|  |  |  | 19- Lack of good research ideas |
|  |  |  | 20- Lack of ability to publish article after the completion of the project |
|  |  |  | 21- Boring and difficult to research because of the lack of skill |
|  |  |  | 22- Lack of research needs and priorities in university health system |
|  |  |  | 23- Lack of coordination of research priorities with research ideas |
|  |  |  | 24- Inappropriate or insufficient consultation before drafting research proposals |
|  |  |  | 25- Inappropriate or insufficient guidance for writing |
|  |  |  | 26- Lack of research applications in personal life and professional job |
|  |  |  | 27- Lack of interest in research |
|  |  |  | 28- Lack of significant income to do research |
|  |  |  | 29- Lack of confidence in potential for completing research |
|  |  |  | 30- Prefer to use the free time to do other task |
|  |  |  | 31- Fear of making mistakes in research and being blamed by others |
|  |  |  | 32- Lack of professor input with students |
| **student practices towards research** | | | |
| **1-Did you participate in a research project before?**  A) YES B) NO | | | |
| **2-** **how many research project did you participate in? (please write zero if you never participated)**  …………………………………………………………………………………. | | | |
| **3-** **How many publications doyou have? (please write zero if you don’t have publications)**  …………………………………………………………………………………. | | | |
| **4-** **Have you ever enrolled/Attended research methodology workshop or training?**  A) YES B) NO | | | |
| **5- How many poster presentations did you do? (please write zero if you don’t have poster presentation)**  …………………………………………………………………………………. | | | |
| **6- How many Research related oral presentations did you do? (please write zero if you don’t have oral presentation)**  …………………………………………………………………………………. | | | |
| **7-** **which process do you contribute in the research? (Choose multiple answers if you participate in more than one process) (Answer only if you participated in a research project before)**   - The concept of research - Literature review - Writing of the proposal - Real execution - Data entry - Data analysis - Manuscript writing | | | |
| **8-** **Type of research projects conducted?? (Answer only if you participated in a research project before).**   - Case reports, - Basic science projects - Retrospective clinical studies - Prospective clinical studies - Clinical trials - Cross-sectional studies - Review articles (narrative or systematic review or meta-analysis) | | | |
